# Revisiting the concept of a symmetric index of agreement for continuous datasets

**DOI:** 10.1038/srep19401

**Published:** 2016-01-14

**Authors:** Gregory Duveiller, Dominique Fasbender, Michele Meroni

**Affiliations:** 1 European Commission, Joint Research Centre, Ispra (VA), I-21027, Italy

## Abstract

Quantifying how close two datasets are to each other is a common and necessary undertaking in scientific research. The Pearson product-moment correlation coefficient *r* is a widely used measure of the degree of linear dependence between two data series, but it gives no indication of how similar the values of these series are in magnitude. Although a number of indexes have been proposed to compare a dataset with a reference, only few are available to compare two datasets of equivalent (or unknown) reliability. After a brief review and numerical tests of the metrics designed to accomplish this task, this paper shows how an index proposed by Mielke can, with a minor modification, satisfy a series of desired properties, namely to be adimensional, bounded, symmetric, easy to compute and directly interpretable with respect to *r*. We thus show that this index can be considered as a natural extension to *r* that downregulates the value of *r* according to the bias between analysed datasets. The paper also proposes an effective way to disentangle the systematic and the unsystematic contribution to this agreement based on eigen decompositions. The use and value of the index is also illustrated on synthetic and real datasets.

Many applications in most branches of sciences require researchers, analysts and decision-makers to compare different datasets against each other and to judge how close they are from one another. The objective may be to compare simulations coming from different models trying to portray a given phenomenon, to compare the same physical quantity measured by different instruments, or to assess if changes in a given data processing chain is resulting in considerably different results.

The notion of closeness (or “agreement”) is a concept that many mathematical formulations try to capture. Examples include commonly used indicators such as the Pearson product-moment correlation coefficient (*r*), the coefficient of determination (*R*^2^) and the root mean square error (RMSE). Intuitively one may classify as disagreement between two datasets any difference from equality. Graphically, any deviation of the experimental data points from the 1:1 line in the datasets scatterplot. A set of indicators may thus be indeed sufficient to express the “distance” of the available data points from the 1:1 line. For instance, the slope and the offset of a linear model fitting the data and a measure of the dispersion around this line may portrait accurately enough the agreement between two datasets. The development of numerical models further stimulated the need for metrics that could serve for their calibration^1^ or to evaluate their performance^2^. An inconvenience is that most indicators are to be considered simultaneously, complicating the comparison of the agreement among multiple datasets. An elegant graphical solution was put forward by Taylor^3^ to visualize concurrently various metrics that contain complementary information, but this still fails to quantify agreement in a single index. Some authors^4^ proposed a fuzzy logic framework to combine different metrics together into a single indicator that should reflect how an expert perceives the quality of a model performance, but this requires a certain subjectivity in the definition of the membership functions. A single metric that characterises patterns like *r*, yet also includes information on the magnitude of deviations, is of clear interest for many users, as reflected by the various proposals of dedicated indices of agreement in different domains such as hydrology, climatology and remote sensing^5^,^6^,^7^,^8^,^9^.

Index symmetry is an important property when considering assessing dataset agreement. Unlike validation or calibration exercises, where some model estimates are compared to reference values that are considered error-free (usually observations of the quantity of interest), in inter-comparison studies there might not be a reference. Because two datasets being compared have some, often unknown or poorly characterized uncertainty, there is not, *a priori*, one dataset that is “better” than the other. As a consequence, an index to evaluate the agreement between *X* and *Y* datasets should be equal to that calculated between *Y* and *X*, a symmetry requirement often not satisfied by validation metrics. Further aspects that should be considered include the possibility of: (1) reformulating indices to show their relationship with better known metrics, such as *r* or RMSE^10^,^11^; and (2) disentangling systematic from the non-systematic random difference in data agreement^6^. The systematic component can be interpreted as a regularized bias due to known or discoverable factors while the unsystematic element is a random component caused by noise or unknown factors. Differentiating between the two is interesting because the systematic difference can in principle be removed by regression analysis.

In this study we review various metrics proposed in the literature that can serve to assess dataset agreement. We then test and inter-compare their performances over synthetic datasets and thus point out some of their shortcomings. We justify why an permutation based index originally proposed by Mielke^7^ can, after a small modification, be considered as the most appropriate because it satisfies all desired properties for such an index, including that of being interpretable with respect to the coefficient of correlation *r*. We also propose a refined approach to investigate separately the unsystematic and the systematic contributions to the dataset disagreement. Finally, we apply the available metrics and the proposed index to two real inter-comparison study cases: one related to time series of the Normalized Difference Vegetation Index (NDVI) acquired during the same time period by two different satellite missions, and the other related to two time series of gross primary production (GPP) estimated by different modelling approaches.

## Desired properties of an index of agreement

Let us first generalise the problem by stating that we seek an index quantifying the agreements between datasets *X* and *Y*. The datasets are measured in the same units and with the same support. In the case of most geospatialized raster datasets, this would mean that both have the same spatial and temporal resolutions. An optimal index of agreement should be:

- **Dimensionless**. This makes it independent of the unit of measurement. It facilitates the comparison of agreement among different pairs of datasets (if each pair has different units for instance) or within different parameter space (*e.g.* for multi-variable datasets).
- **Bounded** between a lower bound (such as 0) corresponding to no agreement, and an upper bound (such as 1) corresponding to perfect agreement. A corollary is that higher values should always indicate higher agreement.
- **Symmetric**, that is, it should have the same numeric value if the values of 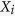 and 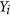 are switched in the equation. This is necessary because of the assumption that, for an assessment on agreement, there is no reference to compare to.
- **Easy to compute** so that it can be used on large datasets.
- **Interpretable** with respect to a widely accepted and familiar measure such the coefficient of correlation *r*.

## Background on existing metrics

Perhaps the most straightforward metric to use to compare two different datasets is the Pearson product-moment correlation coefficient (*r*). It measures the degree of linear dependence between two variables and is expressed as:


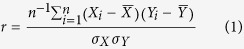


where 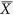 and 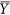 denote the mean values of *X* and *Y*, and 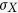 and 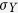 represent their standard deviations. The metric *r* is dimensionless and is bounded between −1 and 1. A value of zero indicates there is no linear dependence between the two variables, while a value of 1 or −1 indicate perfect linear dependence (the latter with a negative dependence).

Another common approach is to consider that a statistical model can be fitted to the data. In this case, a measure of agreement can be inferred from the coefficient of determination 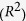, which indicates how well data fit the chosen model. In the case of linear models, the coefficient of determination is equivalent to the square of *r*, and ranges from 0 to 1. Another interesting property is that this number represents the proportion of the variance explained by the model. A disadvantage of both *r* and *R*^2^ is that they only measure the strength of the relationship between the data, but give no indication if the data series have similar magnitude.

To assess if the values of two series match, a metric summarising the deviations between all pairs of values can be computed. A generic form to express such metrics is as follows^12^:


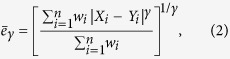


where 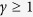 and 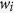 is a weight given to each deviation. If we use equal weights to simplify the notation, when 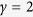 the expression becomes that of the root mean square deviation (RMSD) and when 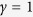, we obtain the mean absolute deviation (MAD). Note that in this context, we prefer referring to these metrics as deviations instead of errors (*i.e.* RMSE and MAE), because we do not consider one dataset to be more correct than the other. With respect to *r* and *R*^2^, RMSD and MAD have the advantage that they provide an information on the absolute differences, or the biases, between *X* and *Y*. A disadvantage is that they are dimensional. To overcome this problem, they are sometimes expressed in percentage by dividing the deviations by one of the two mean values 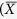 or 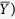. However, this renders the metric asymmetric, and they become unstable when the denominators are small values.

Various indices have been designed to evaluate model skill using the following formulation:


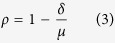


where *δ* is a metric measuring deviations between model estimation and reference observations (such as MAD or the mean square deviation, MSD), and *μ* is a set of reference deviations. The logic behind such formulation is that *μ* should be never be smaller than *δ*, thereby fixing the upper-bound of *ρ* to 1. An example much used in hydrology is known a the coefficient of efficiency^5^ that was later generalized^13^ as:


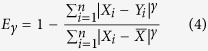


where *X* represents the observations and *Y* the model predictions. Such coefficients of efficiency range from 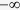 to 1. While the lack of a negative bound may seem an inconvenience, such indices have another practical reference point in that when 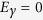, this means that the model is not performing better than by just taking the mean^14^. The original version^5^, for which 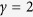, can be additionally be related to the 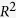
10,11,14. However, such indices are not symmetric, as replacing *X* by *Y* does not lead to the same results.

Willmott^6^ proposed another index for evaluating model performance against measured observations that can be generalized as:


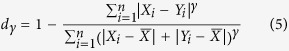


In this case, the denominator *μ* is defined by summing the differences of all points in both *X* and *Y* with respect to 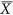, the mean value of *X*. The original version was based on squared deviations 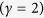, but was later changed^15^ by using absolute deviations arguing that MAD (or in this case MAE since they refer to errors between predictions and observations instead of deviation) is a more natural measure of average error and is less ambiguous than RMSD (or RMSE)^12^. Another refinement of the index^16^ seeked to remove the predictions 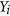 from the denominator, but as argued by others^14^, this amounts to rescaling the expression of the coefficient of efficiency 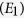 while losing the interesting reference point of 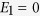. Again, these indices do not respect the symmetry requirement.

Mielke^7^,^17^ proposed another definition of the denominator *μ* based on a non-parametric approach using random permutations. In this case, the baseline consists of the sum of differences between each point and every other point. Such index can be expressed generically for different *γ* values as such:


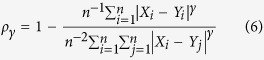


Unlike previous indices, these are symmetric. A disadvantage is that calculating *μ* is computationally expensive, especially when *n* is large. However, for 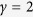 the denominator 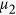 can also be expressed in a simpler form^7^ (a mathematical demonstration is provided in the Supplementary Information) that can be easily computed:


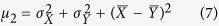


Watterson^8^ proposed to construct an index to evaluate climate model performance by applying an arcsine transformation to Mielke’s 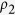 index:


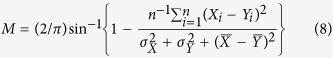


The arcsine function transformation is justified by Watterson as enabling a linear convergence to unity while preserving the original properties of Mielke’s index^8^.

Contrasting with previous studies, Ji & Gallo^9^ explicitly designed an index that would satisfy the symmetry criteria. This index, proposed for inter-comparison of remote sensing imagery, is defined as follows:


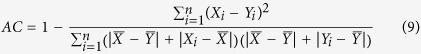


In this case, the baseline of the denominator *μ* is defined based on both 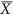 and 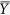. However, this latter index has some serious shortcomings that are illustrated in the following analysis.

## Metric inter-comparison

**Figure 1:**
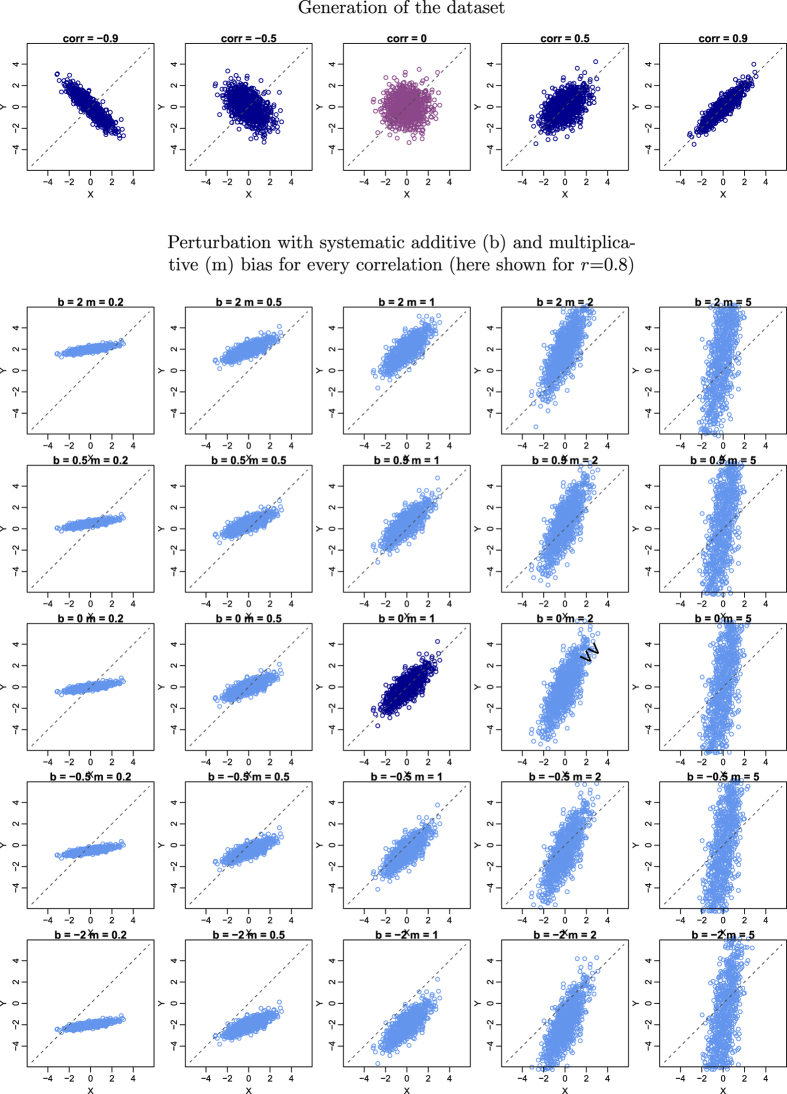
Graphical representation of the generated *X* and *Y* datasets (in the top panel) for a selection of increasing correlations and (bottom panel) after perturbation by either: adding different *b* values to *Y*, rescaling *Y* by a factor *m* or doing both at the same time. In this example the starting *X*–*Y* dataset in the centre corresponds to that having a correlation of *r* = 0.8 (and no additive nor multiplicative bias).

An inter-comparison of metric performance is here proposed for the indices mentioned in section 0 that satisfy the criteria of symmetry: Mielke’s 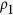 and 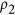 permutation-based indices, Watterson’s *M* index and Ji & Gallo’s agreement coefficient *AC*. To do so, an artificial dataset is produced. Two independent random vectors of 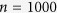 samples with mean of 0 and standard deviation of 1 are first generated and completely decorrelated using a Cholesky decomposition^18^,^19^. These two vectors, 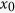 and 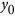, are then recombined together in order to generate two new vectors, 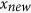 and 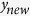, that have a given imposed correlation (the details on how to do this are explained in the Supplementary Information section). The *X* and *Y* datasets are thus generated by respectively aligning together all 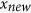 and 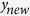 vectors generated for correlations ranging from −1 to 1 (they are illustrated for some correlation values in Fig. 1). Using *X* and *Y*, the agreement metrics can be calculated and compared for pairs of vectors with equal means and variances, but with correlation ranging from −1 to 1. To assess how metrics behave when there is a bias in the data, the *Y* dataset is further perturbed by introducing various systematic additive biases (in practice, by adding *b* to the data) and systematic proportional biases (by multiplying the data by *m*), as illustrated in Fig. 1. These systematic additive and proportional effects can also interact together either compensating or compounding the disagreement.

**Figure 2:**
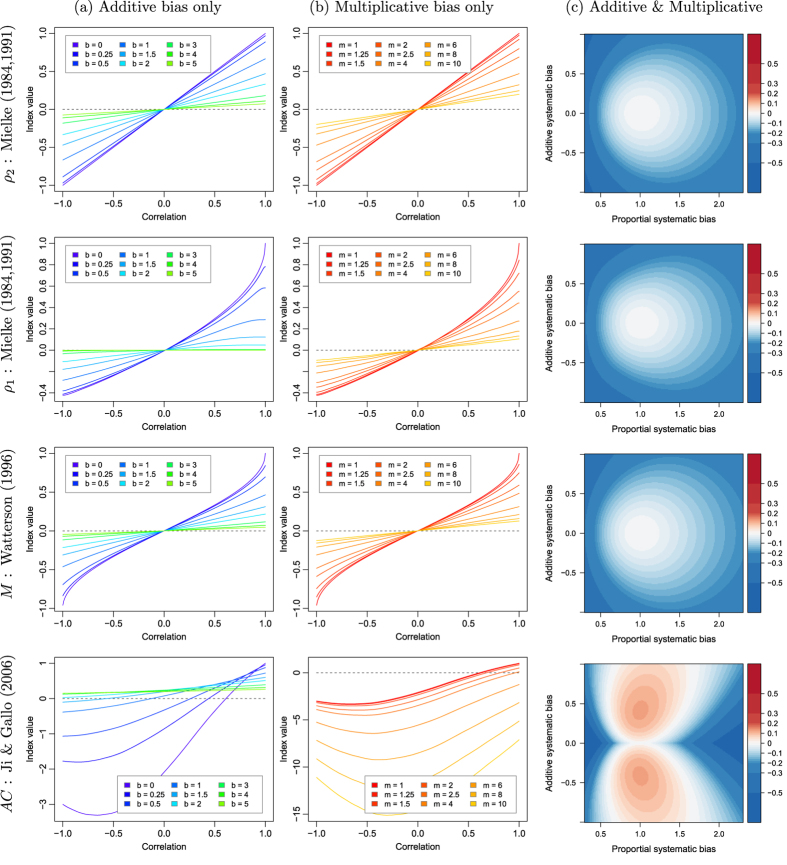
Illustration of how various symmetric agreement metrics discussed in this paper perform over datasets with: (**a**) varying correlation and systematic additive biases; (**b**) varying correlation and systematic multiplicative biases; and (**c**) varying systematic additive and multiplicative biases with correlation fixed to 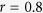. For column (**c**) the values consist of the differences between metrics calculated on a dataset with a given additive and/or proportional systematic bias minus the values calculated on the same datasets but without any bias.

Figure 2 illustrates how the four analysed metrics perform on the generated datasets as a function of imposed additive and multiplicative bias and original correlation between *X* and *Y*. A first remark regarding the plots in columns (a) and (b) of Fig. 2 is that for all metrics, there is an intersection of the iso-lines. The metrics are assumed to correctly portray a decrease in agreement when there is an increased systematic perturbation for all types of correlations. Crossing of these iso-perturbation lines means that this assumption is violated. For *AC* under mild shifts of *b* or rescaling with *m*, abnormal behaviour can be seen even under moderate correlation values (such as with *r* between 0.5 and 0.7). For the 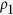, 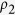 and *M* all lines cross only at 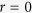. This may be considered to be less inconvenient, as the negative values of the indices could be used to evaluate how much datasets agree in magnitude despite disagreeing in sign. Yet, this adds ambiguity in the interpretation of the index which is not desirable. A second point is that Ji & Gallo’s *AC* is not negatively bounded by zero as it is designed to be. This happens even when no systematic perturbation is added to the data (i.e. when 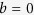 and 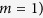 and even for fairly high positive correlation. These are conditions which could be easily expected for most dataset inter-comparison, suggesting how Ji & Gallo’s *AC* should be avoided.

The systematic additive and proportional biases can interact. To illustrate this, column (c) of Fig. 2 shows the value between the index calculated for 2 vectors with a given combination of biases minus the index value calculated from the same vectors without any bias. This is only shown for a given correlation of 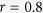. This graphical representation can help illustrate the sensibility of an index to small changes in *b* and *m*. Most indices react similarly, with the notable exception of *AC*. Ji & Gallo’s *AC* index can be higher (*i.e.* more agreement) with a combination of small biases than without any bias at all.

To summarize the outcome of this analysis, it can be stated that all metrics have at least one shortcoming: at some point or another, smaller index values counter-intuitively represent higher agreement. For all of them, it is also unclear how they can be related to the coefficient of correlation. Additionally, Ji & Gallo’s *AC* has strongly undesired behaviour in the presence (but also in the absence) of bias. While Mielke’s 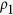 index is computationally expensive, the 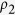 index, with its simplified expression, appears to be a suitable candidate for dataset comparisons when the correlation is zero or positive. However, from the mathematical formulation proposed by the author it is not explicit how 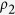 is related to the coefficient of correlation. We believe that this latter point deserves further investigation because the user of the index will typically have a clear understanding of what a correlation value means, but will not be familiar the values taken by agreement index itself.

## Rationale for choosing the right metric

If we can accept the use an index based on MSE rather than MAE, we argue that the correct metric to choose should be a slightly modified version of Mielke’s 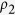 index. This argumentation stems from the idea that for an index constructed based on the structure of equation (3), the objective should be to define the denominator *μ* as the maximum value that the numerator *δ* can take. Finding the smallest value that maximizes the numerator (i.e. its supremum) is important in order to ensure having an index with the maximum possible sensitivity. For MSE based indices, it can be shown (see Supplementary Information) that the numerator can be rewritten as:


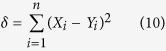



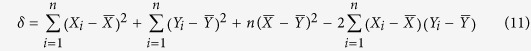


where the last term is proportional to the covariance between *X* and *Y*. One way to ensure 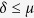 is to create an index 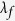 containing this covariance term explicitly in the denominator and constrain it to always be positive:


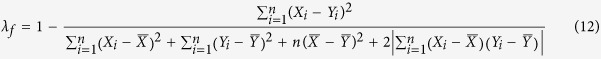


**Figure 3:**
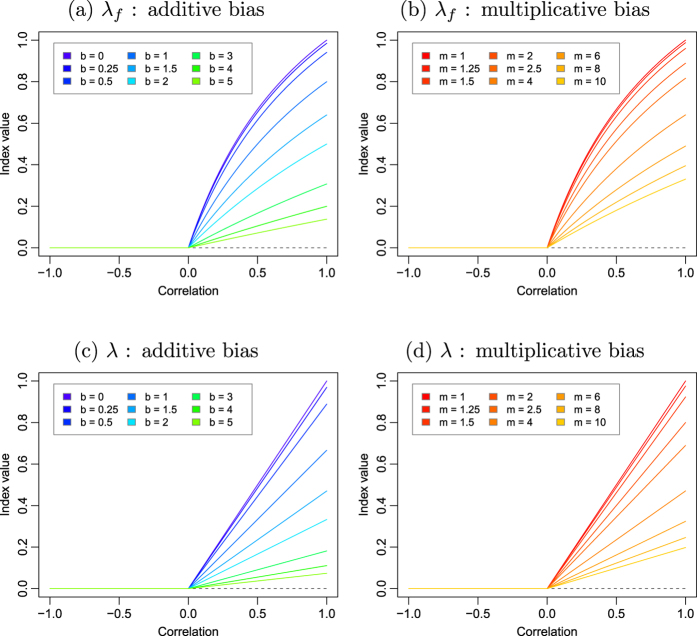
Illustration of how the indices of agreement *λ*_*f*_ and *λ* discussed in this paper perform over datasets with varying correlation and systematic additive or multiplicative biases.

The four terms in the denominator can be represented geometrically as illustrated in the Supplementary Information section. As a result of adding the covariance term explicitely, the index 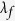 ensures that when *X* and *Y* are negatively correlated, 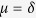, resulting in an index equal to zero when 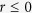 as can be seen in Fig. 3. However, when 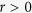 the denominator is needlessly inflated by this covariance term, as the numerator will always be smaller due to the negative sign in front of the covariance term in equation (10). To solve this issue, we propose to define the index, which we refer to simply as *λ*, as follows:


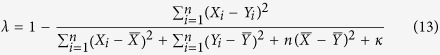


where


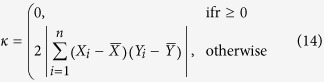


By recognizing the expression of the variances in equation (13), it can be rewritten more succinctly as:


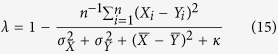


revealing the similarity with equation (7) and the simplified expression of Mielke’s 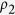 permutation index. As shown in Fig. 3, the resulting index is identical to 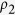 for positively correlated vectors while remaining at 0 when 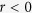.

The index has the additional desirable property that, when 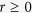 and when there is no additive nor multiplicative bias, it takes the value of the correlation coefficient. If there is a bias, the index will take a lower value than *r* according to a multiplicative coefficient *α* that can only take a value between 0 and 1. Using equation (10), it can effectively be demonstrated (see Supplementary Information) that:


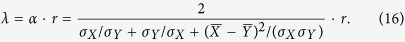


The advantage of this property is that the index value can be immediately compared to *r*, which is a metric most practitioners are familiar with. Any deviation from *r* indicates an increase in bias proportional to *α*.

## Separating the unsystematic from the systematic contribution

Willmott^6^,^20^ proposed that his indices of agreement could provide further insight by separating the effects due to the systematic from the unsystematic components of the deviations. This idea can be generalized to any index formulated using equation (3) by decomposing the deviations into their systematic and unsystematic components as 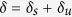 and then defining new systematic and unsystematic indices respectively as 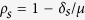 and 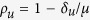. The unsystematic index, 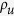, can be interpreted as the value that the standard index would take if all bias is disregarded, which therefore relates to the noise around a line passing through the data. The systematic index, 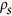, is more difficult to grasp. A better way to understand the information it contains is presenting it as the proportion of deviations composed of systematic noise 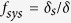.

To calculate these new derived indices, the relationship between *X* and *Y* must first be characterized, which then allows the computation of 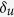, and finally that of 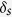 by subtracting 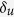 from *δ*. The theoretical relationship between *X* and *Y* is assumed to be linear: 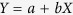. Willmott^6^ uses an ordinary least-square regression to estimate *a* and *b*. This may be acceptable when the *X* dataset is considered to be a reference, but not when trying to establish agreement without assuming a reference because of a violation of the symmetry between *X* and *Y*, *i.e.* a regression of *X* on *Y* is not equivalent to that of *Y* on *X*. To solve this issue, Ji & Gallo^9^ propose to use a geometric mean functional relationship (GMFR) model^21^,^22^, for which *b* and *a* are derived as follows:


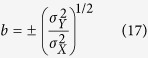



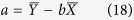


the sign of *b* being the one of the correlation coefficient. While solving the problem of symmetry, this approach is far from optimal because of how *b* is defined. The problem is illustrated in the case in which two totally uncorrelated dataset with variances of 2 and 4 will still have a positive slope of 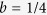 instead of a flat line.

Once the relationship between *X* and *Y* is characterised, the second question is how to represent the deviations from that line. Willmott^6^ uses the sum of square deviations in the *Y* axis:


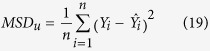


where 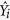 is obtained from the regression 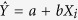. Ji & Gallo^9^ use the sum of product-difference:


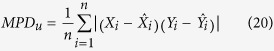


where both 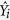 and 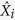 need to be obtained from the GMFR regression. Both these approaches have the same flaw. In order to be coherent with the definition of the total deviations, the unsystematic deviations should be calculated orthogonally from the 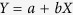 line *i.e.* as if the 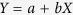 line would be the 1:1 line.

We propose a solution to solve both problems (*i.e.* the definition of the line and the calculation of the deviations orthogonally) by working with the principal planes of the *X*-*Y* cloud. By applying an eigen decomposition to the covariance matrix of *X* and *Y*, we obtain the two eigenvectors describing the principal axes of the cloud of points. The first eigenvector can serve to define the line of the principal axis in the *X*-*Y* space (*i.e.* the 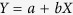 line). The second eigenvector can be used to calculate the vector *h* containing the distances of all *X*-*Y* points orthogonally from this principal axis (for more details on how to calculate the eigenvectors, the resulting 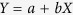 line of the principal axis and the vector *h*, see the Supplementary Information). The unsystematic mean squared deviation can then be calculated based on the *h* distances as follows:


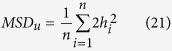


**Figure 4:**
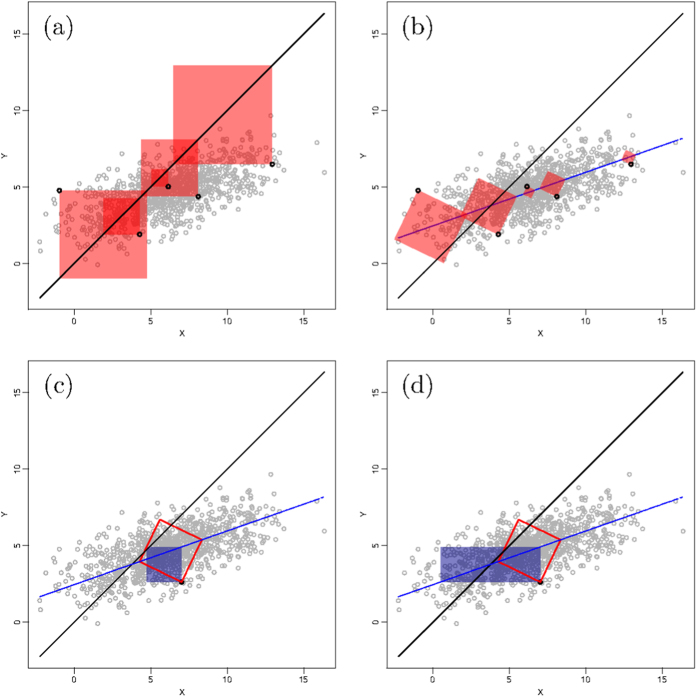
Geometrical representation of different characterisation of deviations: (**a**) the total squared deviations for a selection of points (represented by the surface of the red squares); (**b**) the unsystematic squared deviations for the same points calculated orthogonally to the principal axis of the cloud point as proposed in this paper; (**c**) comparison (for one point only to ensure clarity) of how such deviation (red empty square) differs from what is suggested by Willmott^6^ (dark solide square); and (**d**) similar comparison with the product-difference proposed by Ji & Gallo^9^ (dark solid rectangle).

Figure 4 provides a geometric representation of how these unsystematic squares differ from the total squares calculated with respect to the 1:1 line. Figure 4 also illustrates geometrically how these unsystematic squares differ in surface from what is proposed by Willmott^6^ and Ji & Gallo^9^ using the formulations of equation (19) and (20).

An important remark regarding the unsystematic index 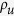 is that, although it disregards any bias, it does not mean it is equivalent to the correlation. Neither is 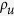 equivalent to *α* in the case of *λ*. The difference can be appreciated by considering a point cloud and rotating it. This alters their correlation, but thanks to the eigen decomposition, 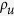 will remain the same. For the case of *λ*, this also results in positive values for 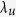 when 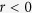, which can be interpreted as a measure of noise in the data.

In case the deviations *δ* are to be characterized by absolute differences instead of squared differences (for indices such as 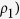, the equivalent unsystematic mean absolute deviation can also be calculated from *h* as follows:


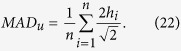


Yet another option could be to use directly the distance values of *h* as a measure of deviations.

## Demonstration for real applications

To illustrate how the proposed index can be used in real case studies and how it compares with other metrics, some examples using actual data are provided. Geophysical datasets are typically structured based on the familiar 2 or 3 spatial dimensions, plus the temporal dimension, resulting in time series of geospatial data. It is often interesting to evaluate separately the temporal evolution of the spatial agreement and the spatial patterns of temporal agreement with dedicated protocols^23^. For the sake of brevity a limited analysis is provided here, in which the first example focuses on temporal agreement of time series of satellite imagery, while the second example illustrates spatial agreement of different modelled gross primary productivity (GPP) for a single moment in time.

## Temporal comparison of two satellite observation datasets

The first study case consists of satellite measurements of the Normalized Difference Vegetation Index (NDVI) obtained from 1 October 2013 up to 31 May 2014 over North-West Africa. The spatial resolution is 1 km and the temporal resolution is a dekad (a dekad is a period resulting of the division of each calendar month into 3 parts, which can thus take values of 8, 9, 10 or 11 days). Data is obtained by two different instruments on-board of two different satellite platforms: SPOT-VEGETATION and PROBA-V (these will be respectively referred to as VT and PV for simplicity). PV data are available from the Copernicus Global Land Service portal^24^, while VT archive data are provided courtesy of the JRC MARSOP project^25^. Although the geometrical and spectral characteristics of the satellites and the processing chains of the data have been designed to be as close as possible, differences between the products are still expected because the instruments are not the same. The interest here is to quantify where in the region do the time series disagree. Since there is no grounds to argue one should be a better reference than the other, a symmetric index of agreement should be applied on each pair of time series, resulting in values that can be mapped spatially.

**Figure 5:**
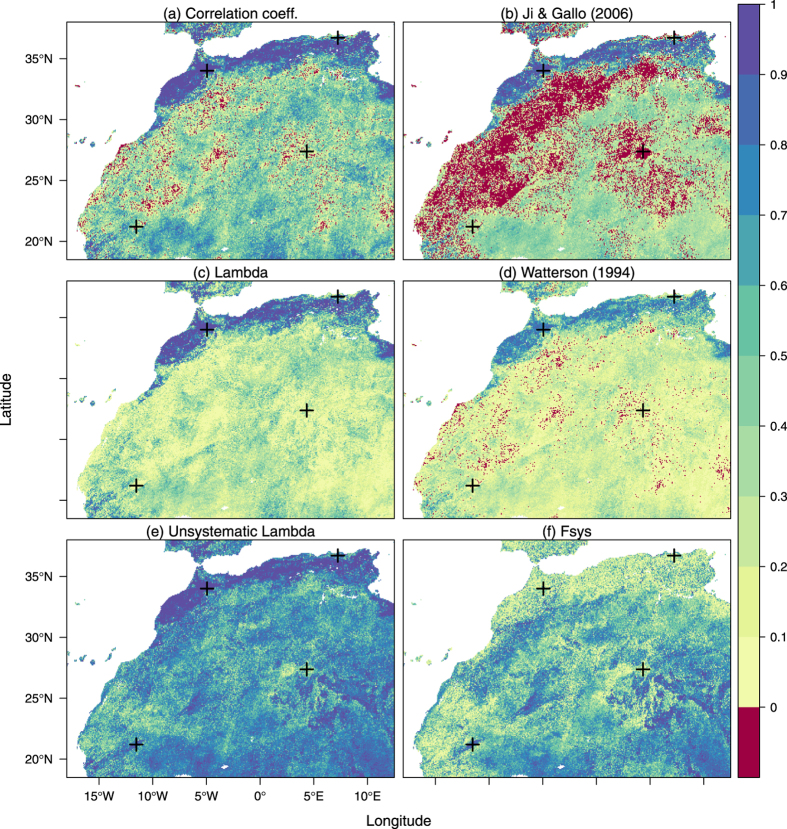
Spatialisation of the temporal agreement between time series coming from two Earth observation satellites according to various agreement metrics described in the text, calculated and plotted with the R statistical software (version 3.2.1, http://www.R-project.org/). The crosses represent the spatial location of the time series depicted in Fig. 6.

Results are presented in the maps of Fig. 5. All maps show the expected patterns of temporal agreement: areas with a strong dynamic NDVI signal, such as the Northern cultivated areas, have a higher agreement than desert areas where the signal is mostly composed of noise. However, there is a large difference in where each metric provides negative values: The *λ* map shows no negative values, the map of Watterson’s *M* metric takes negative values only were the correlation is negative, but the map of Ji & Gallo’s *AC* index shows vast areas of negative values throughout the area. The comparison between *λ* and *r* reflects the added-value in using the former, which incorporates the biases not present in the latter. The magnitude of these biases with respect to the total deviations can be spatialized in the 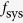 map, while the agreement of the datasets irrespective of these biases is shown in the 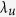 map.

**Figure 6:**
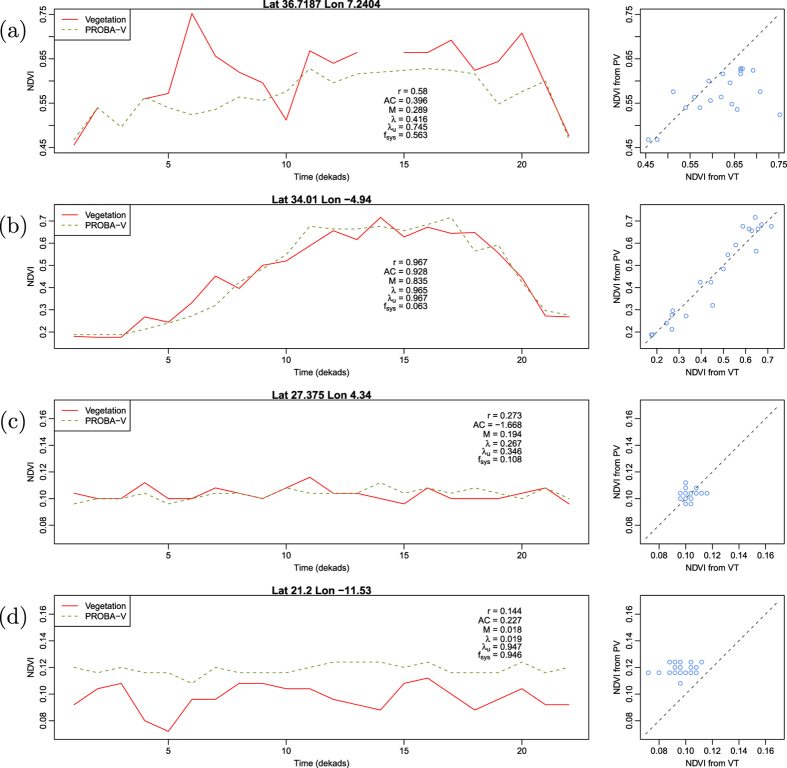
Comparison of time series of measurement done by two different Earth observation satellites. The location of each time series in the study zone can be seen in Fig. 5.

From the selected time series shown in Fig. 6, one can better appreciate differences in the numerical value of the different metrics with respect to the analysed data (temporal profiles and scatterplots). By focusing on the profiles of Fig. 6c,d collected over very arid areas, the better performances of *λ* as compared to *AC* are clear. Both profiles show limited temporal variability and reduced correlation. *AC* shows a negative (and out of bounds) value of −1.668 for profile c and a positive and higher value (0.227) for profile d that has a lower correlation and a clear bias. *λ* instead shows value lower than the two correlations and decrease for profile d as compared to c. Interestingly enough, 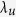 for profile d informs us that by applying a linear transformation, the agreement would be greatly improved.

## Spatial comparison of two geophysical products

The second case study consists of estimations of gross primary productivity (GPP) at global scale for a given moment in time: June 2007. The first dataset is the NASA MOD17 product^26^, produced by combining canopy biophysical information derived from satellite remote sensing products with meteorological and land cover information, with some adjustments based on localised *in situ* flux-tower observations. The product used is a version that has been aggregated to a spatial resolution of 0.05° and a monthly temporal resolution (available here:^27^). The second dataset is the MPI MTE-GPP product^28^ constructed using a decision tree statistical approach to upscale information from flux-towers up to a 0.5° grid, aided by gridded meteorological and remote sensing co-variables (available here:^29^). Both approaches employ similar sets of information but using considerably different methodologies, and again, there is no reason to consider one is better than the other.

**Figure 7:**
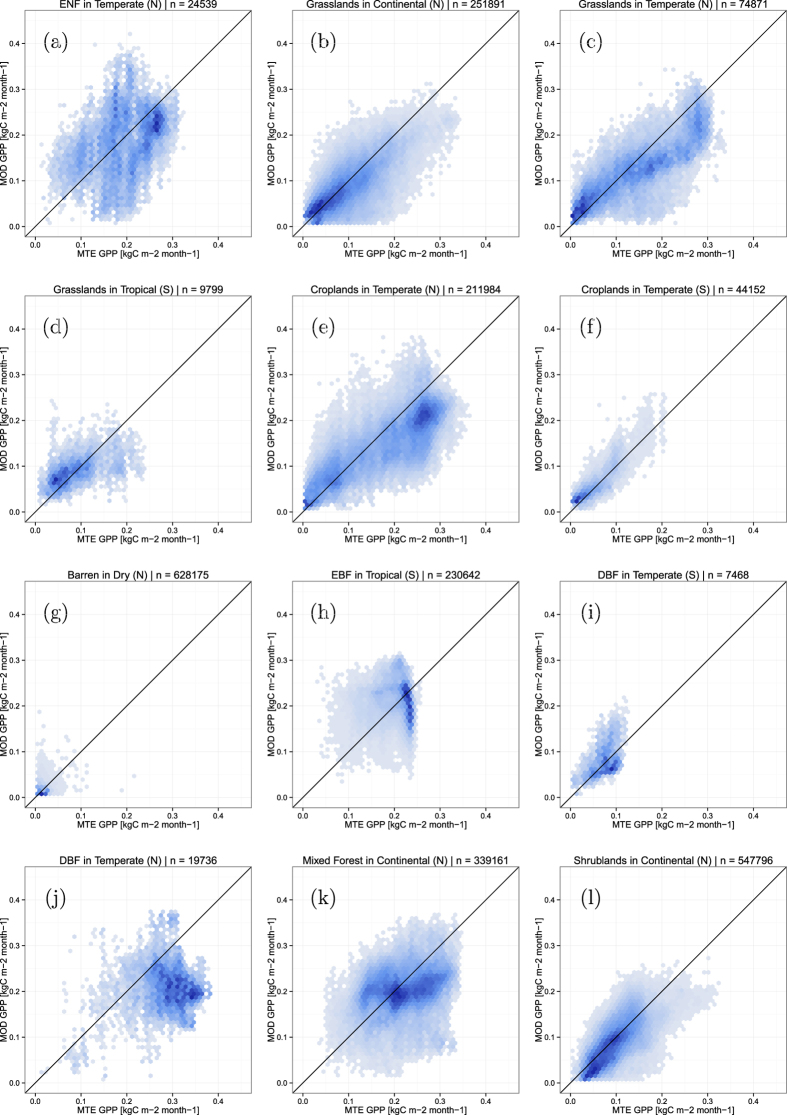
Selection of cases comparing two global gross primary productivity products for different land cover types across different climate zones in either the northern (N) or southern (S) hemispheres. ENF, EBF and DBF stand respectively for evergreen needleleaf, evergreen broadleaf and deciduous broadleaf forests.

The products are to be compared spatially for sets of equal reasoning areas. These areas are defined by a combination of climatic zones^30^ and land cover types (based on the MODIS 2007 MCD12C1 Land cover product^31^). All pixels within a given area in one product are compared to the corresponding pixels of the other product. The results are summarized as 2-D histograms in Fig. 7 and Table 1. The examples shown include 6 cases in which the *AC* value is below zero. As can be understood from the index definition (eq. (9)) and from the plots in Fig. 7, this typically occurs when the means are very similar while the variances are strongly dissimilar. The examples also show how *M* is systematically lower than *λ* (as expected due to its arcsine transformation), which makes the result less comparable to *r*. Good examples include evergreen needleleaf forest (case a) and shrublands (case l) which are very close to having no bias, and thus *α* close to 1, but *M* considerably lower than *r*. Three cases (g,h and j) also illustrate how the proposed index takes a value of 0 when 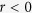, but 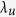 does not. Finally, high values of 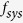 (such as case j) help to quickly identify when the scatter clouds are not on the 1:1 line.

## Concluding remarks

Through numerical evaluation of different proposed metrics, this paper shows that a modified version of the index of Mielke is preferable to others. This index, named here *λ*, is adimensional, bounded, symmetric, easy to compute and directly interpretable with respect to the commonly used Pearson coefficient of correlation *r*. This index can basically be considered as a natural extension to *r* that downregulates the value of *r* according to the bias encountered in the data.

The logic behind the original conception of the index by Mielke^7^, based on all the possible permutations between the elements within the two datasets, intuitively suggests how its denominator is indeed the maximum possible value that the mean sum of squares can attain. Because of the mathematical properties of these squared deviations, it is possible to rewrite this index in an expression based on variances instead of permutations, making it much simpler to compute. Unfortunately, we have not succeeded to generalize the structure of the (easily computable) index to be used with other metrics of deviations, such as the mean absolute deviations. However, the demonstration of how to disentangle the unsystematic from the systematic contribution in the agreement using an eigen decomposition could be applied to any other type of metric.

The scope of the index remains to be a pragmatic extension of *r* and thus used in a context where a linear functional agreement is wanted. It is not intended as a tool to explore new functional associations in the data (such as the maximum information coefficient^32^). However, its use could go beyond comparing dataset agreement symmetrically, and join the collection of existing methods^2^ to characterize model performance with respect to a reference. Also, the index was demonstrated here with case studies of spatio-temporal gridded data, but it should also be usable for any pair of vectors of any kind of data, just as *r*.

## Additional Information

**How to cite this article**: Duveiller, G. *et al.* Revisiting the concept of a symmetric index of agreement for continuous datasets. *Sci. Rep.* **6**, 19401; doi: 10.1038/srep19401 (2016).

## Supplementary Material

Supplementary Information

## Figures and Tables

**Table 1:** Agreement between two global gross primary productivity products for different land cover types across different climate zones in either the northern (N) or southern (S) hemispheres.

	Zone	*r*	*AC*	*M*	*λ*	*λ*_*u*_	*f*_*sys*_	*α*
(a)	ENF inTemperate (N)	0.37	−0.49	0.24	0.36	0.40	0.07	0.98
(b)	Grasslands inContinental (N)	0.75	0.53	0.52	0.72	0.76	0.12	0.97
(c)	Grasslands inTemperate (N)	0.87	0.70	0.59	0.80	0.88	0.41	0.93
(d)	Grasslands inTropical (S)	0.57	−0.20	0.34	0.51	0.67	0.32	0.90
(e)	Croplands inTemperate (N)	0.84	0.67	0.54	0.75	0.87	0.47	0.89
(f)	Croplands inTemperate (S)	0.88	0.78	0.66	0.86	0.89	0.20	0.97
(g)	Barren inDry (N)	−0.09	−0.41	−0.05	0.00	0.21	0.27	0.00
(h)	EBF inTropical (S)	−0.14	−2.59	−0.09	0.00	0.34	0.42	0.00
(i)	DBF inTemperate (S)	0.45	−0.27	0.28	0.43	0.54	0.19	0.94
(j)	DBF inTemperate (N)	−0.01	0.21	−0.00	0.00	0.68	0.68	0.00
(k)	Mixed Forest inContinental (N)	0.28	−0.14	0.16	0.25	0.42	0.22	0.90
(l)	Shrublands inContinental (N)	0.77	0.57	0.53	0.74	0.78	0.14	0.97

ENF, EBF and DBF stand respectively for evergreen needleleaf, evergreen broadleaf and deciduous broadleaf forests.
