# Author Correction: Revisiting the concept of a symmetric index of agreement for continuous datasets

**DOI:** 10.1038/s41598-022-23771-z

**Published:** 2022-11-22

**Authors:** Gregory Duveiller, Dominique Fasbender, Michele Meroni

**Affiliations:** Joint Research Centre, European Commission, 21027 Ispra, VA Italy

Correction to: *Scientific Reports* https://doi.org/10.1038/srep19401, published online 14 January 2016

This Article contains an error in Equation 15, where the $\kappa$ in the denominator is not multiplied by ${n}^{-1}$.

$$\lambda =1-\frac{{n}^{-1}{\Sigma }_{i=1}^{n}({X}_{i}-{Y}_{i}{)}^{2}}{{\sigma }_{X}^{2}+{\sigma }_{Y}^{2}+(\overline{X}-\overline{Y}{)}^{2}+\kappa }$$

should read:

$$\lambda =1-\frac{{n}^{-1}{\Sigma }_{i=1}^{n}({X}_{i}-{Y}_{i}{)}^{2}}{{\sigma }_{X}^{2}+{\sigma }_{Y}^{2}+(\overline{X}-\overline{Y}{)}^{2}+{n}^{-1}\kappa }$$

An extra note of caution is warranted regarding the variances ${\sigma }_{X}^{2}$ and ${\sigma }_{Y}^{2}$ in the denominator. These are population variances (${\sigma }^{2}=\frac{\Sigma ({x}_{i}-\overline{x})}{n}$) and not sample variances (${S}^{2}=\frac{\Sigma ({x}_{i}-\overline{x})}{n-1}$). Using ${S}^{2}$ instead of ${\sigma }^{2}$ would lead to slight discrepancies.

