# Supplementary material for: Revisiting the concept of a symmetric index of agreement for continuous datasets
Source: Sci Rep. 2016 Jan 14;6:19401. doi: 10.1038/srep19401 (PMC4725897; doi:10.1038/srep19401)
Supplement: Supplementary Information [file srep19401-s1.pdf]

Supplementary information for: Revisiting the concept of a symmetric index of agreement for continuous datasets

Gregory Duveiller[1,*], Dominique Fasbender[1], and Michele Meroni[1]

[1]European Commission, Joint Research Centre, Ispra (VA), I-21027, Italy
[*]gregory.duveiller@jrc.ec.europa.eu

ABSTRACT

This supplementary information sections provides further information on the mathematical operations and demonstrations relevant to this study, in order to support and simplify the text in the main paper.

1 Generating data series with a given correlation structure

In the study, it is necessary to generate data series with a given correlation structure. To do so, it is necessary to start with series that are completely decorrelated. When two vectors are randomly and independently generated, there is very little chance that they will result in having zero correlation. If $\mathbf{X}$ is a matrix containing two such randomly generated (and zero-centred) vectors, the problem consists in finding the matrix $\mathbf{A}$ that can be used to transform $\mathbf{X}$ into a new matrix $\mathbf{Y}$ containing completely uncorrelated vectors:

$$\mathbf{Y} = \mathbf{A}\mathbf{X} \tag{1}$$

The correlation between the vectors in matrices $\mathbf{X}$ and $\mathbf{Y}$ is characterized by their respective covariance matrices Σ_X and Σ_Y. For $\mathbf{Y}$ to be completely uncorrelated, Σ_Y must equal the identity matrix $\mathbf{I}$. A property of linear composition of random vectors such as equation (1) is that their covariance matrices are related as such:

$$\Sigma_Y = \mathbf{A}\Sigma_X\mathbf{A}^T \tag{2}$$

where $\mathbf{A}^T$ is the transpose of $\mathbf{A}$. The problem can thus be reformulated as finding $\mathbf{A}$ that solves:

$$\Sigma_Y = \mathbf{I} = \mathbf{A}\Sigma_X\mathbf{A}^T \tag{3}$$

The solution is $\mathbf{A} = \mathrm{Chol}(\Sigma_X^{-1})$ where Σ_X^{-1} is the inverse matrix of Σ_X and Chol() is an operator providing the result of a Cholesky decomposition. The Cholesky decomposition consists in the factorization of a matrix $\mathbf{M}$ (which must be a positive-definite Hermitian matrix) into an upper triangular matrix $\mathbf{U}$ that satisfies this relationship:

$$\mathbf{M} = \mathbf{U}^T\mathbf{U} \tag{4}$$

The following demonstration shows how setting $\mathbf{A} = \mathrm{Chol}(\Sigma_X^{-1})$ in equation (2) results in uncorrelated vectors:

$$\Sigma_Y = \mathrm{Chol}(\Sigma_X^{-1})\Sigma_X\,\mathrm{Chol}(\Sigma_X^{-1})^T \tag{5}$$

$$\Sigma_Y\,\mathrm{Chol}(\Sigma_X^{-1}) = \mathrm{Chol}(\Sigma_X^{-1})\Sigma_X\,\mathrm{Chol}(\Sigma_X^{-1})^T\,\mathrm{Chol}(\Sigma_X^{-1}) \tag{6}$$

$$\Sigma_Y\,\mathrm{Chol}(\Sigma_X^{-1}) = \mathrm{Chol}(\Sigma_X^{-1})\Sigma_X\Sigma_X^{-1} \tag{7}$$

$$\Sigma_Y\,\mathrm{Chol}(\Sigma_X^{-1}) = \mathrm{Chol}(\Sigma_X^{-1})\mathbf{I} \tag{8}$$

$$\Sigma_Y = \mathbf{I} \tag{9}$$

Equation (1) can also be used to generate a matrix containing vectors with an imposed correlation structure defined by a covariance matrix Σ_{new}. If matrix $\mathbf{X}$ is completely uncorrelated, by setting $\mathbf{A} = \mathrm{Chol}(\Sigma_{new})^T$ in equation (2) it can be shown

that the covariance of the new matrix $\mathbf{Y}$ is Σ_{new}:

$$\Sigma_Y = \mathrm{Chol}(\Sigma_{new})^T \Sigma_X \, \mathrm{Chol}(\Sigma_{new}) \tag{10}$$
$$= \mathrm{Chol}(\Sigma_{new})^T \mathbf{I} \, \mathrm{Chol}(\Sigma_{new}) \tag{11}$$
$$= \mathrm{Chol}(\Sigma_{new})^T \, \mathrm{Chol}(\Sigma_{new}) \tag{12}$$
$$= \Sigma_{new} \tag{13}$$

The two operations of decorrelating and then imposing a correlation can be done in a single step by using this equation:

$$\mathbf{X}_{new} = \mathrm{Chol}(\Sigma_{new})^T \, \mathrm{Chol}(\Sigma_X^{-1})\mathbf{X} \tag{14}$$

2 A simplified expression of the permutation of squared differences

The permutation of squared differences used in the index proposed by Mielke can be rewritten in terms of variances and squared bias, thus facilitating its computation:

$$\sum_{i=1}^{n} n^{-1} \sum_{j=1}^{n} n^{-1} (X_i - Y_j)^2 = n^{-2} \sum_{i=1}^{n} \sum_{j=1}^{n} (X_i^2 - 2X_iY_j + Y_j^2) \tag{15}$$
$$= n^{-2} \{ n \sum_{i=1}^{n} X_i^2 - 2 \sum_{i=1}^{n} X_i \sum_{j=1}^{n} Y_j + n \sum_{j=1}^{n} Y_j^2 \} \tag{16}$$
$$= n^{-1} \sum_{i=1}^{n} X_i^2 + n^{-1} \sum_{j=1}^{n} Y_j^2 - 2 \cdot n^{-1} \sum_{i=1}^{n} X_i \cdot n^{-1} \sum_{j=1}^{n} Y_j \tag{17}$$
$$= n^{-1} \sum_{i=1}^{n} X_i^2 + n^{-1} \sum_{j=1}^{n} Y_j^2 - 2\bar{X}\bar{Y} \tag{18}$$

Because $\sum_{i=1}^{n} (X_i - \bar{X})^2$ can be shown to be equal to $\sum_{i=1}^{n} X_i^2 - n\bar{X}^2$, the first two terms of the right side of the equation above can respectively be replaced by $n^{-1} \sum_{i=1}^{n} (X_i - \bar{X})^2 + \bar{X}^2$ and $n^{-1} \sum_{j=1}^{n} (Y_j - \bar{Y})^2 + \bar{Y}^2$ to result in:

$$\sum_{i=1}^{n} n^{-1} \sum_{j=1}^{n} n^{-1} (X_i - Y_j)^2 = n^{-1} \sum_{i=1}^{n} (X_i - \bar{X})^2 + \bar{X}^2 + n^{-1} \sum_{j=1}^{n} (Y_j - \bar{Y})^2 + \bar{Y}^2 - 2\bar{X}\bar{Y} \tag{19}$$
$$= n^{-1} \sum_{i=1}^{n} (X_i - \bar{X})^2 + n^{-1} \sum_{j=1}^{n} (Y_j - \bar{Y})^2 + \bar{X}^2 - 2\bar{X}\bar{Y} + \bar{Y}^2 \tag{20}$$
$$= n^{-1} \sum_{i=1}^{n} (X_i - \bar{X})^2 + n^{-1} \sum_{j=1}^{n} (Y_j - \bar{Y})^2 + (\bar{X} - \bar{Y})^2 \tag{21}$$
$$= \sigma_x^2 + \sigma_y^2 + (\bar{X} - \bar{Y})^2 \tag{22}$$

3 Rewriting the sum of squares

The sum of squared differences can be rewritten as four terms related to the variance in X, the variance in Y, the mean deviation and the covariance between X and Y:

$$\sum_{i=1}^{n}(X_i - Y_i)^2 = \sum_{i=1}^{n}(X_i - Y_i + \bar{X} - \bar{X} + \bar{Y} - \bar{Y})^2 \tag{23}$$

$$= \sum_{i=1}^{n}((X_i - \bar{X}) - (Y_i - \bar{Y}) + (\bar{X} - \bar{Y}))^2 \tag{24}$$

$$= \sum_{i=1}^{n}(X_i - \bar{X})^2 + \sum_{i=1}^{n}(\bar{Y} - Y_i)^2 + n(\bar{X} - \bar{Y})^2 \tag{25}$$

$$-2\sum_{i=1}^{n}(X_i - \bar{X})(Y_i - \bar{Y}) - 2\sum_{i=1}^{n}(Y_i - \bar{Y})(\bar{X} - \bar{Y}) \tag{26}$$

$$+2\sum_{i=1}^{n}(\bar{X} - \bar{Y})(X_i - \bar{X}) \tag{27}$$

$$= \sum_{i=1}^{n}(X_i - \bar{X})^2 + \sum_{i=1}^{n}(Y_i - \bar{Y})^2 + n(\bar{X} - \bar{Y})^2 - 2\sum_{i=1}^{n}(X_i - \bar{X})(Y_i - \bar{Y}) \tag{28}$$

This last expression can also be divided by n to rewrite it more simply as:

$$n^{-1}\sum_{i=1}^{n}(X_i - Y_i)^2 = \sigma_x^2 + \sigma_y^2 + (\bar{X} - \bar{Y})^2 - 2\,\mathrm{Cov}(X,Y) \tag{29}$$

When considering the index λ_f defined using the expression as follows:

$$\lambda_f = 1 - \frac{\sum_{i=1}^{n}(X_i - Y_i)^2}{\sum_{i=1}^{n}(X_i - \bar{X})^2 + \sum_{i=1}^{n}(Y_i - \bar{Y})^2 + n(\bar{X} - \bar{Y})^2 + 2\left|\sum_{i=1}^{n}(X_i - \bar{X})(Y_i - \bar{Y})\right|} \tag{30}$$

the various terms in the numerator and the denominator can be represented geometrically as illustrated in Fig. S1.

4 How to relate the index of agreement to the correlation coefficient

The expression of the index of agreement described in this paper can be rearranged to explicitly relate to the correlation coefficient when $r \geq 0$ and $\kappa = 0$.

$$\lambda = 1 - \frac{n^{-1}\sum_{i=1}^{n}(X_i - Y_i)^2}{\sigma_x^2 + \sigma_y^2 + (\bar{X} - \bar{Y})^2} \tag{31}$$

$$= \frac{\sigma_x^2 + \sigma_y^2 + (\bar{X} - \bar{Y})^2 - n^{-1}\sum_{i=1}^{n}(X_i - Y_i)^2}{\sigma_x^2 + \sigma_y^2 + (\bar{X} - \bar{Y})^2} \tag{32}$$

The numerator of the right side of equation (32) is equal to $2\,\mathrm{Cov}(X,Y)$, as shown by rearranging terms of equation (29). This simplifies the notation to:

$$\lambda = \frac{2\,\mathrm{Cov}(X,Y)}{\sigma_x^2 + \sigma_y^2 + (\bar{X} - \bar{Y})^2} \tag{33}$$

$$= \frac{2\,\mathrm{Cov}(X,Y)}{\sigma_x^2 + \sigma_y^2 + (\bar{X} - \bar{Y})^2} \cdot \frac{1/(\sigma_X\sigma_Y)}{1/(\sigma_X\sigma_Y)} \tag{34}$$

$$= \frac{2}{\sigma_X/\sigma_Y + \sigma_Y/\sigma_X + (\bar{X} - \bar{Y})^2/(\sigma_X\sigma_Y)} \cdot \frac{\mathrm{Cov}(X,Y)}{\sigma_X\sigma_Y} \tag{35}$$

$$= \alpha \cdot r \tag{36}$$

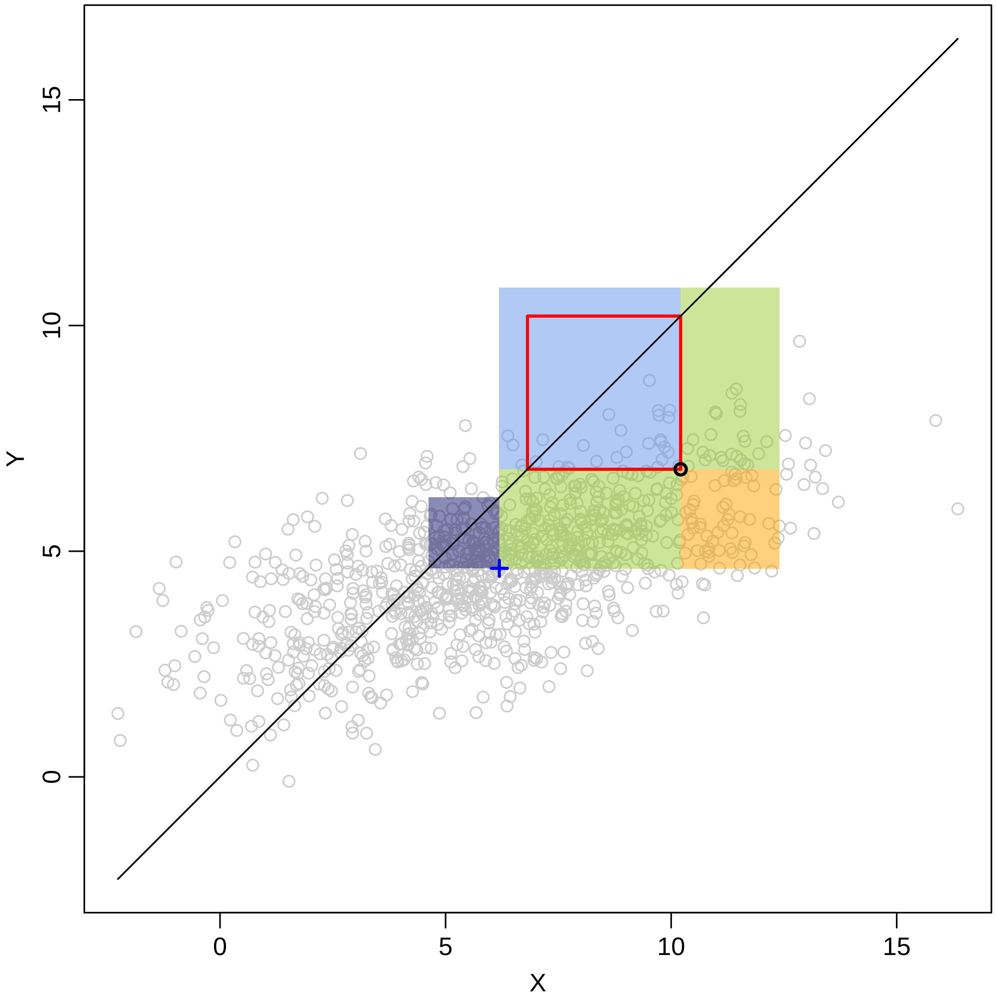

Figure S1. Illustration of the how different terms in the definition of λ_f can be represented geometrically for a given point (the black circle) in the data cloud whose centre is shown with the blue cross: (a) the empty red square represents the numerator $(X_i - Y_i)^2$; (b) the light blue and yellow squares represent the respective deviations in X and Y, that is $(X_i - \bar{X})^2$ and $(Y_i - \bar{Y})^2$; (c) the dark blue square represents the third term in the denominator $(\bar{X} - \bar{Y})^2$; and (d) the two green rectangles correspond to the surfaces represented in last term as: $(X_i - \bar{X})(Y_i - \bar{Y})$.

18 ## 5 How to express the line obtained by eigen decomposition

The proposition to separate the unsystematic from the systematic part of the agreement between two data series X and Y involves using an eigen decomposition of their covariance matrix. The eigen decomposition consists in factorizing a square matrix $\mathbf{M}$ into a matrix $\mathbf{P}$ of eigenvectors and a diagonal matrix $\mathbf{D}$ containing eigenvalues:

$$\mathbf{M} = \mathbf{P}\mathbf{D}\mathbf{P}^T \tag{37}$$

If the covariance matrix of the couple (X,Y) is defined as:

$$\Sigma = \begin{pmatrix} \sigma_X^2 & \sigma_{XY} \\ \sigma_{YX} & \sigma_Y^2 \end{pmatrix} \tag{38}$$

and assuming that $\sigma_{XY} \neq 0$, then the eigenvalues λ_1 and λ_2 of Σ can be expressed as:

$$\lambda_1 = \frac{\sigma_X^2 + \sigma_Y^2 + \sqrt{(\sigma_X^2 + \sigma_Y^2)^2 - 4\det\Sigma}}{2} \tag{39}$$

and

$$\lambda_2 = \frac{\sigma_X^2 + \sigma_Y^2 - \sqrt{(\sigma_X^2 + \sigma_Y^2)^2 - 4\det\Sigma}}{2} \tag{40}$$

The corresponding eigenvectors $\mathbf{v_1}$ and $\mathbf{v_2}$ are then defined as:

$$\mathbf{v}_1 = \frac{1}{\sqrt{1 + \left(\frac{\lambda_1 - \sigma_X^2}{\sigma_{XY}}\right)^2}} \begin{pmatrix} 1 \\ \frac{\lambda_1 - \sigma_X^2}{\sigma_{XY}} \end{pmatrix} \tag{41}$$

and

$$\mathbf{v}_2 = \frac{1}{\sqrt{1 + \left(\frac{\lambda_1 - \sigma_X^2}{\sigma_{XY}}\right)^2}} \begin{pmatrix} \frac{\lambda_1 - \sigma_X^2}{\sigma_{XY}} \\ -1 \end{pmatrix} \tag{42}$$

From the first eigenvector $\mathbf{v_1}$, which defines the principal axis of the point cloud of (X,Y), the slope of the $Y = a + bX$ line describing this axis can be obtained:

$$b = \frac{\lambda_1 - \sigma_X^2}{\sigma_{XY}} \tag{43}$$

Once b is known, the intercept a can be calculated based on the mean values of X and Y:

$$a = \bar{Y} - b\bar{X} \tag{44}$$

The second eigenvector $\mathbf{v_2}$ can be used to calculate the orthogonal distances h of all points from the $Y = a + bX$ as follows:

$$h = [\mathbf{XY}]\mathbf{v_2} \tag{45}$$

19 Note that in the case that $\sigma_{XY} = 0$, the eigenvectors are simply parallel to the X and Y axes.
